# Extract from an Article on Dysentery among Negroes

**Published:** 1854-10

**Authors:** Samuel A. Cartwright


### RECORD OF MEDICAL SCIENCE.

*Extract from an article on Dysentery among Negroes.*
BY SAMUEL A. CARTWRIGHT, M. D.

On a large sugar plantation on the coast, belonging to Capt. Wm. J. Minor, where the cholera appeared about two years ago, a removal of the negroes from one set of houses to another set, (one of which had been built for the purpose,) was twice tried without arresting the progress of the disease at all. At length, after forty had died, being called on to visit the plantation, and invested with full power to do as I pleased, I took about three hundred negroes, sick and well, a mile or two back into a dry, open place in the swamp, where there was no house to be seen, or any preparation begun for building any. The overseer refused to obey until I recorded this fact in the plantation book, with an additional statement over my own signature, that a cloud was coming up, and in all probability there would be rain. Having made the record, I headed the line of march, and, sure enough, the rain came up and all got wet. They encamped in the open air and built fires, although the weather was warm, and some booths were directed to be made over the sick to protect them from the sun and the rain. The ashy-colored, dry skin conjurers, or prophets, who had alarmed their fellow-servants with the prophesies that the cholera was to kill them all, and who had gained, by various tricks and artifices, much influence over their superstitious minds, were by my orders, at twilight, called up, stripped, and greased with fat bacon, in presence of the whole camp—a camp without tents or covering of any kind, except some bushes and boards over the sick from the carts that conveyed them to the camp. After being greased, the grease was well slapped in with broad leather straps, marking time with the *tam tam*, a wild African dance that was going on in the centre of the camp among all those, who had the physical strength to participate in it. This procedure drove the cholera out of the heads of all who had been conjured into the belief that they were to die with that disease; because it broke the charm of the conjurers by converting them, under the greasing and slapping process, into subjects for ridicule and laughter, instead of fear and veneration. The next morning, by times, all who had been able to join in the dance the over night, were ordered into the cane-field to work. There were no more cases of cholera, or deaths from that disease after the removal, except one man who had strayed away from the camp, and except also among some half dozen who had been left to take care of the houses, about half of whom died. The removal included not only the negroes, but the horses, mules and dogs, that there might be no excuse for revisiting the houses and haunts of civilized life. They remained in the camp at night, and labored in the fields by day for some six weeks before they were brought back to the houses, and during all that time they enjoyed good health. —*New Orleans Med. and Sur. Jour.*

